# Supplementary material for: Positional Behavior of Introduced Monk Parakeets (Myiopsitta monachus) in an Urban Landscape
Source: Animals (Basel). 2022 Sep 11;12(18):2372. doi: 10.3390/ani12182372 (PMC9494974; doi:10.3390/ani12182372)
Supplement: Supplementary file 1 [file animals-12-02372-s001.zip › animals-1890713-supplementary.pdf]

Supplemental Tables

Supplementary Table S1. Overall percentage (%) of positional behaviors collected from an established colony of Quaker parrots (*Myiopsitta monachus*) from Brooklyn, New York City during the study period. Percentages of locomotor repertoire calculated for all behaviors and for non-aerial (i.e., excluding flight and hovering) behaviors.

	**Locomotion**	
	**All**	**Non-aerial**
Flight	67.51	na
Hover	4.40	na
Flight_short_interbranch	11.05	39.34
Bridge	0.08	0.27
Climb	1.30	4.64
Climb_beak-assisted	0.92	3.28
Climb_wing-assisted	0.90	3.19
Descent	0.41	1.46
Descent_beak-assisted	0.33	1.18
Descent_wing-assisted	0.46	1.64
Hop	0.72	2.55
Leap	0.20	0.73
Leap_wing-assisted	2.38	8.47
Run	0.20	0.73
Sidling	3.20	11.38
Suspensory	0.10	0.36
Uprighting	0.13	0.46
Walk	5.70	20.31
Number of observations	3909	1098
	Posture	
Cantilevering	0.23	
Cling	0.37	
Hang	0.19	
Perch	96.93	
Stand	2.28	
Number of observations	7337	

Supplemental Table S2. Overall percentage (%) of weather conditions and the associated positional behaviors collected from an established colony of Quaker parrots (*Myiopsitta monachus*) from Brooklyn, New York City during the study period. No substrate or orientation code was scored if the animals' feet were not in contact with the support (e.g., flight, hovering).

	**Overcast**	**Rain**	**Snow**	**Sun**
Pooled	26.27	4.79	0.10	68.84
Number of observations	2954	539	11	7742
		Locomotion		
Flight	61.70	80.90	100.00	68.36
Hover	6.04	2.51	0.00	4.00
Flight_short_interbranch	12.84	7.04	0.00	10.79
Bridge	0.00	0.00	0.00	0.11
Climb	2.05	1.01	0.00	1.08
Climb_beak-assisted	0.86	2.51	0.00	0.83
Climb_wing-assisted	1.08	0.00	0.00	0.90
Descent	0.54	0.00	0.00	0.40
Descent_beak-assisted	0.32	0.00	0.00	0.36
Descent_wing-assisted	0.97	0.00	0.00	0.32
Hop	1.19	1.51	0.00	0.51
Leap	0.22	0.00	0.00	0.22
Leap_wing-assisted	7.34	3.02	0.00	0.69
Run	0.00	0.00	0.00	0.29
Sidling	3.13	0.00	0.00	3.46
Suspensory	0.00	0.00	0.00	0.14
Uprighting	0.22	0.00	0.00	0.11
Walk	1.51	1.51	0.00	7.43
Number of observations	927	199	11	2772
		Posture		
Cantilevering	0.30	0.00	0.00	0.22
Cling	0.54	2.65	0.00	0.14
Hang	0.15	0.00	0.00	0.22
Perch	98.57	97.35	0.00	96.24
Stand	0.44	0.00	0.00	3.18
Number of observations	2027	340	0	4970

Supplemental Table S3. Overall percentage (%) of substrate type and the associated positional behaviors observed on these substrates collected from an established colony of Quaker parrots (*Myiopsitta monachus*) from Brooklyn, New York City during the study period. No substrate or orientation code was scored if the animals' feet were not in contact with the support (e.g., flight, hovering).

	**Ground**	**Very large**	**Large**	**Medium**	**Small**	**Terminal**	**Nest**	**Artificial**
Pooled	4.05	0.21	0.45	5.43	14.70	19.80	44.76	10.60
Number of observations	342	18	38	458	1240	1670	3776	894
Locomotion								
Flight	na	na	na	na	na	na	na	na
Hover	na	na	na	na	na	na	na	na
Flight_short_interbranch	0.00	50.00	0.00	23.61	33.33	52.84	64.54	18.99
Bridge	0.00	0.00	0.00	2.78	0.48	0.00	0.00	0.00
Climb	0.00	0.00	0.00	2.78	2.86	0.57	10.25	6.33
Climb_beak-assisted	0.00	50.00	14.29	0.00	4.29	0.57	4.43	6.33
Climb_wing-assisted	0.00	0.00	0.00	0.00	0.00	0.00	8.31	6.33
Descent	0.00	0.00	0.00	4.17	1.90	1.14	1.94	0.00
Descent_beak-assisted	0.00	0.00	14.29	0.00	0.95	1.70	1.39	2.53
Descent_wing-assisted	0.00	0.00	0.00	0.00	2.38	0.00	2.22	6.33
Hop	5.95	0.00	14.29	4.17	2.86	0.00	1.11	3.80
Leap	0.00	0.00	14.29	0.00	2.38	0.00	0.00	2.53
Leap_wing-assisted	0.00	0.00	0.00	5.56	7.14	38.07	0.00	8.86
Run	2.70	0.00	0.00	0.00	0.95	0.00	0.00	1.27
Sidling	0.00	0.00	0.00	40.28	35.24	3.41	3.60	3.80
Suspensory	0.00	0.00	0.00	0.00	0.95	0.57	0.00	1.27
Uprighting	0.00	0.00	0.00	0.00	1.43	0.00	0.55	0.00
Walk	91.35	0.00	42.86	16.67	2.86	1.14	1.66	31.65
Number of observations	185	8	7	72	210	176	361	79
Posture								
Cantilevering	0.00	0.00	0.00	0.52	0.00	0.00	0.44	0.00
Cling	0.00	60.00	16.13	0.00	0.00	0.00	0.23	0.98
Hang	0.00	0.00	0.00	0.00	0.68	0.20	0.12	0.00
Perch	0.00	40.00	83.87	98.96	99.32	99.80	99.21	98.04
Stand	100.00	0.00	0.00	0.52	0.00	0.00	0.00	0.98
Number of observations	157	10	31	386	1029	1494	3416	815

Supplemental Table S4. Overall percentage (%) of sampling effort during the study period and the associated positional behaviors observed during each month collected from an established colony of Quaker parrots (*Myiopsitta monachus*) from Brooklyn, New York City during the study period. No substrate or orientation code was scored if the animals' feet were not in contact with the support (e.g., flight, hovering).

	**January**	**February**	**March**	**April**	**May**	**June**
Pooled	1.52	24.79	29.88	10.38	12.60	20.84
Number of observations	171	2789	3361	1168	1417	2344
Locomotion						
Flight	76.92	69.58	51.65	63.35	81.50	76.71
Hover	0.00	3.08	6.03	8.74	2.97	2.90
Flight_short_interbranch	3.85	8.87	18.37	13.11	6.11	7.04
Bridge	0.00	0.37	0.00	0.00	0.00	0.00
Climb	0.00	0.74	2.83	0.73	0.35	0.93
Climb_beak-assisted	0.00	1.11	1.10	0.24	0.70	1.04
Climb_wing-assisted	0.00	1.23	1.10	0.00	0.00	1.35
Descent	0.00	0.00	0.18	0.24	1.40	0.52
Descent_beak-assisted	1.92	0.49	0.46	0.00	0.17	0.21
Descent_wing-assisted	0.00	0.86	0.37	0.00	0.00	0.72
Hop	0.00	1.48	0.27	0.00	1.22	0.62
Leap	0.00	0.37	0.18	0.00	0.35	0.10
Leap_wing-assisted	5.77	4.80	4.66	0.00	0.00	0.00
Run	0.00	0.00	0.09	0.00	0.35	0.52
Sidling	7.69	4.19	3.29	5.58	0.52	2.59
Suspensory	0.00	0.00	0.09	0.00	0.00	0.31
Uprighting	0.00	0.12	0.18	0.00	0.17	0.10
Walk	3.85	2.71	9.14	8.01	4.19	4.35
Number of observations	52	812	1094	412	573	966
Posture						
Cantilevering	0.00	0.05	0.22	0.00	0.59	0.44
Cling	3.36	0.81	0.26	0.53	0.12	0.00
Hang	0.00	0.10	0.49	0.13	0.00	0.00
Perch	96.64	98.48	97.35	98.54	93.36	95.07
Stand	0.00	0.56	1.68	0.79	5.92	4.50
Number of observations	119	1977	2267	756	844	1378

Supplemental Table S5. Overall percentage (%) of substrate-orientation and the associated positional behaviors observed on these orientations collected from an established colony of Quaker parrots (*Myiopsitta monachus*) from Brooklyn, New York City during the study period. No substrate or orientation code was scored if the animals' feet were not in contact with the support (e.g., flight, hovering).

	**Horizontal**	**Oblique**	**Vertical**
Pooled	69.24	25.64	5.12
Number of observations	5841	2163	431
Locomotion			
Flight	na	na	na
Hover	na	na	na
Flight_short_interbranch	29.93	42.14	60.38
Bridge	0.18	0.63	0.00
Climb	0.70	3.14	17.45
Climb_beak-assisted	0.70	1.89	12.26
Climb_wing-assisted	5.81	0.00	0.94
Descent	0.00	1.89	4.72
Descent_beak-assisted	0.18	1.57	3.30
Descent_wing-assisted	2.29	0.94	0.94
Hop	3.87	1.89	0.00
Leap	1.23	0.31	0.00
Leap_wing-assisted	3.17	23.58	0.00
Run	1.41	0.00	0.00
Sidling	13.38	15.41	0.00
Suspensory	0.53	0.31	0.00
Uprighting	0.88	0.00	0.00
Walk	35.74	6.29	0.00
Number of observations	568	318	212
Posture			
Cantilevering	0.06	0.00	6.39
Cling	0.02	0.16	10.50
Hang	0.13	0.38	0.00
Perch	96.62	99.46	83.11
Stand	3.17	0.00	0.00
Number of observations	5273	1845	219
